# Co-occurring Conditions and Quality of Life in Autistic Children Attending General Education or Special Education Schools

**DOI:** 10.1007/s10803-025-06839-3

**Published:** 2025-05-07

**Authors:** Chantal van den Helder, Rachel Plak, Sander Begeer, Martijn Meeter

**Affiliations:** 1 Department of Clinical, Neuro and Developmental Psychology, Faculty of Behavioural and Movement Sciences, Vrije Universiteit Amsterdam and Amsterdam Public Health, Van Der Boechorststraat 7, 1081 BT Amsterdam, The Netherlands; 2 Institute of Education and Child Studies, Faculty of Social and Behavioural Sciences, Leiden University, Leiden, The Netherlands; 3 LEARN! Research Institute, Vrije Universiteit Amsterdam, Amsterdam, The Netherlands

**Keywords:** Autism, Children, Co-occurring conditions, Quality of life, Education

## Abstract

A majority of children with autism is diagnosed with one or more co-occurring mental health conditions. We aimed to explore how co-occurring conditions and Quality of Life differ between school types (general education vs. special education schools), and whether co-occurring conditions were subject to the same trends over time for the two school types. Secondary, we aimed to analyze associations with child characteristics. Repeated cross- sectional data from 2013 to 2022 on children with autism (N = 1534,aged 5–15 years) in the Netherlands were used. Across school types, we showed a decrease in externalizing and language-learning conditions over time. Language-learning conditions became more concentrated in special education schools. Hyperactivity-inattention difficulties remained stable, emotional difficulties decreased and behavioral difficulties increased. Compared to those in general education, children in special education schools faced more behavioral and hyperactivity-inattention challenges. School type, duration of the study, externalizing, internalizing and language-learning conditions were all unrelated to QoL. Autistic girls experienced more behavioral difficulties compared to boys with autism. In conclusion, children with autism in special education schools experience similar QoL, but have more (severe) co-occurring conditions than those attending general education.

**Supplementary Information:**

The online version contains supplementary material available at https://doi.org/10.1007/s10803-025-06839-3.

## Co-occurring Conditions and Quality of Life in Autistic Children Attending General Education or Special Education Schools

A majority of children with autism, approximately 70% (Simonoff et al., 2008), are diagnosed with one or more co-occurring psychiatric disorders (American Psychiatric Association, 2013; Hossain et al., 2020; Lord et al., 2022; McCauley et al., 2020). A range of factors including intelligence, age, gender, autistic traits and social economic status may be linked to this trajectory, which in turn can could be related to the quality of life (QoL) (Colvert et al., 2022; Leader et al., 2021; Lord et al., 2022; Louwerse et al., 2015; McCauley et al., 2020; Simonoff et al., 2013, 2020). Children spend a considerable part of their day in school environments, which likely appears to be related to the onset and intensity of co-occurring conditions and ultimately their QoL (Green, 2019; Menezes & Mazurek, 2021; Schneider et al., 2022). Despite the shift in the western world towards inclusive education, the role of special education schools remains indispensable, as they provide smaller classes, specialized teachers and enhanced support tailored for an autism-friendly environment, making them a crucial resource for children with more complex disabilities (Ledoux et al., 2020; Morningstar et al., 2017; Rattaz et al., 2020; Van den Helder et al., 2024). Additionally, within inclusive education teachers tend to be less positive towards the inclusion of (autistic) children with behavioral, or severe emotional difficulties or specific learning disabilities in general education (De Boer et al., 2011; Krischler & Pit-ten Cate, 2020; Russell et al., 2023; Tant & Watelain, 2016). This may coincide with a concentration of co-occurring conditions among children with autism in special education schools during inclusive education (Rattaz et al., 2020). Challenges in school can arise, such as following instructions or carrying out assignments (Green, 2019; Llanes et al., 2020) particularly when co-occurring conditions are present. Co-occurring conditions have been shown to be related to school attendance, adjustment, daily functioning and QoL (Adams et al., 2020; Fleming et al., 2020; Lord et al., 2022; Menezes & Mazurek, 2021; Schneider et al., 2022). Given the complexity of these challenges, creating a well-adjusted learning environment is crucial for improving the QoL of autistic children (Green, 2019). Despite this, it remains unclear whether special education schools, which may offer more targeted support, are indeed better suited to enhance the QoL of autistic children with co-occurring conditions, as this has yet not been examined.

During key transitions, such as school entry or adolescence, the prevalence and severity of co-occurring conditions like emotional, behavioral, ADHD related, and language-learning difficulties often fluctuate in children with autism (Colvert et al., 2022; Hollocks et al., 2022; Lai et al., 2019; Llanes et al., 2020; McCauley et al., 2020; Simonoff et al., 2013; Stringer et al., 2020). Several studies among children with autism in the UK (aged 4 to 13) and the US (aged 12 to 23) have shown that hyperactivity/inattention and behavioral difficulties tend to increase in severity between ages 4 to 7, followed by a decrease in the subsequent years. Emotional difficulties follow a similar severity trajectory, but tend to stabilize after childhood (Colvert et al., 2022; McCauley et al., 2020; Simonoff et al., 2013; Stringer et al., 2020).

This relative peak in difficulties at age 4 to 7 coincides with the period when children start school. Changes in the frequency and severity of difficulties may align with changes in the school environment. Llanes et al. (2020) concluded in their study that emotional and ADHD difficulties increased in the year after school entry. In contrast, the severity of language-learning conditions has been shown to decrease during the school period (Ibrahim, 2020), including among children in special education schools (Mattison et al., 2022). Despite these findings studies on how school type, whether general education or special education school, relates to the trajectory of co-occurring conditions and problems are scarce. A follow- up study in the UK by Simonoff et al. (2013) among 81 autistic youth aged between 12 and 16, showed a stronger reduction in conduct problems and special education school placement; changes in emotional and hyperactivity problems were not related to school type. In general Stringer et al. (2020) found similar results for these difficulties in a UK study among 158 autistic participants aged 12 to 23. However, conduct problems were not associated with school type. This inconsistency in results may have been influenced by different age ranges analysed in the two studies.

Autistic children who experienced emotional or behavioral difficulties in childhood also experience these problems in adolescence, with greater stability of emotional difficulties in youth with autism than in neurotypical youth (Colvert et al., 2022; Hollocks et al., 2022; Stringer et al., 2020). ADHD conditions and traits exhibit varying prevalence patterns. In most cases, prevalence decreased over time, however some trajectories indicated stability or even an increase. These differences are likely influenced by variations in ages of the sample, as distinguishing ADHD from autism is more challenging in younger children (Hollocks et al., 2022; Llanes et al., 2020; Stringer et al., 2020; Verheij et al., 2015).

Most of these studies on the trajectories of co-occurring conditions have relatively small sample sizes (less than 200) with the risk of overgeneralization of small sample study results. To better understand the risk factors in the trajectory of co- occurring conditions larger studies with more diversity in child characteristics are necessary. Additionally, there is a gap in research exploring the trajectory of co- occurring conditions of autistic youth in different school types. Understanding the relation between school type and these conditions is essential for evaluating the impact on QoL and the fluctuations in these conditions.

Several child characteristics are related to the frequency and severity of co-occurring conditions in children with autism: In many cases, severity in co- occurring conditions increases with age (Lord et al., 2022), with internalizing conditions in autistic girls particularly increasing in adolescence (Gotham et al., 2015). Higher severity of autism traits is positively associated with both the frequency and severity of co- occurring conditions (Colvert et al., 2022; Jang & Matson, 2015). Intelligence, particularly verbal IQ, is positively associated with the severity of internalizing and externalizing conditions (Gotham et al., 2015; McCauley et al., 2020; Salazar et al., 2015;). The association between parental education level and the severity of emotional difficulties in autistic children is unclear (Salazar et al., 2015; Stringer et al., 2020). Results on the relation between gender and co-occurring conditions in children with autism are inconsistent. Some studies found no gender differences in externalizing or internalizing difficulties (Lecavalier et al., 2019; Mayes et al., 2020), potentially due to the small number of females in the samples. However, other studies suggest that autistic boys may be at higher risk for ADHD related difficulties (Bougeard et al., 2021; Salazar et al., 2015), while over time, autistic girls may show a higher risk for severity increase of internalizing difficulties (Gotham et al., 2015). This is consistent with findings from general population studies, which indicate that male participants are more likely to report externalizing conditions and female participants are more likely to report internalizing conditions (Supekar et al., 2017).

The first aim was to examine differences in the frequency and severity of co-occurring conditions and the quality of life (QoL) among autistic students across different school types and years, as well as to assess whether these differences varied over time depending on school type.

Second, we aimed to investigate the associations between the frequency and severity of co-occurring conditions, QoL, and key characteristics of autistic youth—including age, gender, IQ, autistic traits, and family socioeconomic status—while accounting for the effects of school type, year, and their interaction. We anticipated that children with autism in special education schools would have more co-occurring conditions and difficulties than those in general education schools. In addition, we expected that this difference to increase over time. Furthermore, we expected that placement in special education schools would be positively associated with QoL, particularly for children with co-occurring conditions. Finally, we anticipated that child characteristics are related to the trajectory of co-occurring conditions and to the QoL of autistic children. Specifically, we expected age, autistic traits, IQ and socio-economic status would be positively related to the prevalence and severity of co-occurring conditions. Furthermore, we expected that boys would be more likely to exhibit ADHD related symptoms and externalizing conditions or difficulties, whereas girls would be more likely to exhibit internalizing conditions or difficulties.

## Method

### Participants

Repeated cross sectional data from 2013 to 2022 were obtained from the Netherlands Autism Register (NAR) (https://www.nederlandsautismeregister.nl/english/), a database including online parental reports as well as sociodemographic data of autistic children.

Demographic information of participants is shown in Table 1. Participants included adult caregivers of autistic children aged 5 to 15 years (N = 1534), most of them with at least a BA (69%), who reported on their child by filling in questionnaires from the NAR. All children received a formal autism spectrum diagnosis according to the DSM-4 and DSM-5 criteria (American Psychiatric Association, 1994, 2013). Caregivers reported the diagnosis given at a professional location by a qualified clinician unaffiliated with this study. More than half of the children in this sample attended a special education school (56%) There was a decrease in sample size over time (990 participants in 2013 versus 145 in 2022).

**Tabel 1:** Demographic information per wave

Year	Age				Gender		Autistic traits				Intelligence		School type	
					Female						Normal		Special	
	*N*	Mean	*SD*	Min–max	*N*	%	*N*	Mean	*SD*	Min–max	*N*	%	*N*	%
2013	990	11.37	2.82	5.02–15.99	990	19.01	465	81.03	10.56	45.00–108.00	985	39.29	850	56.00
2015	583	12.13	2.46	5.08–15.99	583	20.24	495	81.02	10.43	45.00–108.00	582	39.00	500	59.80
2016	369	12.58	2.35	5.36–15.99	369	21.14	281	81.72	10.28	55.00–105.00	368	37.77	315	57.46
2017	399	12.29	2.63	5.08–15.91	399	21.30	286	82.26	10.78	55.00–108.00	396	34.60	326	59.51
2018	377	12.17	2.62	5.54–15.97	377	23.34	277	81.38	10.79	51.00–106.00	337	33.83	313	56.23
2019	320	12.23	2.73	5.06–15.98	320	22.81	246	81.06	10.76	51.00–106.00	293	37.88	274	57.66
2020	315	11.75	2.87	5.12–15.99	315	26.03	235	79.91	11.21	48.00–107.00	286	39.86	249	52.21
2021	301	11.94	2.64	5.34–15.97	301	25.91	238	81.01	10.15	50.00–106.00	280	40.00	231	47.62
2022	145	12.01	2.36	6.32–15.98	145	26.21	119	80.59	9.98	48.00–106.00	133	33.83	112	50.00
Total	3799	11.95	2.68	5.02–15.99	3799	21.82	2642	81.15	10.57	45.00–108.00	3660	37.87	3170	56.15

Year	Migration background		Family SES				Co-occurring conditions				Quality of Life			
								Externalizing	Internalizing	Language-learning				
	*N*	Yes %	*N*	High %	Middle %	Low %	*N*	Yes %	Yes %	Yes %	*N*	Mean	*SD*	Min–max
2013	990	10.20	551	64.43	29.22	6.35	990	23.9	5.8	10.9				
2015	583	8.58	582	64.95	29.21	5.84	583	24.9	7.5	13.9				
2016	369	8.67	334	67.66	28.14	4.19	369	17.6	4.9	7.9	332	6.58	1.43	1.00–10.00
2017	399	9.77	366	66.39	28.14	5.46	399	21.6	7.0	8.5	311	6.56	1.40	1.00–10.00
2018	377	10.61	350	69.43	26.86	3.71	377	19.1	4.0	8.5	280	6.70	1.51	2.00–10.00
2019	320	8.13	307	68.73	27.36	3.91	320	20.6	8.8	9.7	262	6.71	1.36	2.00–10.00
2020	315	10.48	293	70.31	27.30	2.39	315	14.3	7.0	4.4	177	6.99	1.29	3.00–9.00
2021	301	9.30	296	74.66	23.99	1.35	301	17.3	7.0	8.3	227	6.63	1.43	2.00–9.00
2022	145	13.10	143	72.03	26.57	1.40	145	23.4	13.8	8.3	145	6.51	1.55	2.00–10.00
Total	3799	9.69	3222	67.85	27.78	4.38	3799	21.1	6.7	9.6	1734	6.66	1.43	1.00–10.00

Year	Severity of co-occurring difficulties											
	Emotional difficulties				Behavioral difficulties				Hyperactivity-inattention difficulties			
	*N*	Mean	*SD*	Min–max	*N*	Mean	*SD*	Min–max	*N*	Mean	*SD*	Min–max
2013												
2015												
2016												
2017	263	4.81	2.65	.00–10.00	263	2.16	1.96	.00–8.00	263	4.18	2.15	.00–8.00
2018	283	4.49	2.95	.00–10.00	283	2.15	1.87	.00–9.00	283	4.52	2.23	.00–8.00
2019												
2020	126	4.22	2.76	.00–10.00	126	2.07	1.75	.00–8.00	126	4.77	2.17	.00–8.00
2021												
2022	145	2.23	1.78	.00–9.00	145	4.61	2.72	.00–10.00	145	4.52	2.07	.00–8.00
Total	817	4.15	2.80	.00–10.00	817	2.58	2.26	.00–10.00	817	4.45	2.17	.00–8.00

### Design

The current study has a longitudinal design including nine years of data collected in the Netherlands. Approval for this study was granted by the Ethics Committee of the Vrije Universiteit Amsterdam (VCWE-2020-041R1).

### Measures

#### Co-occurring Conditions

Participants were presented with a list of 25 mental health conditions, which were chosen based on their frequency of occurrence in previous studies. In the Dutch healthcare system, co-occurring diagnoses are provided according to DSM-5 criteria, and parents were asked to report their child’s current co-occurring DSM-5 diagnoses or conditions as identified at a professional location (American Psychiatric Association, 2013). Responses were recoded into three categories: internalizing conditions (mood disorders, anxiety disorders, post-traumatic stress disorders and burn out problems), externalizing conditions (ADHD, behavioral disorder (ODD/CD) and language-learning conditions (speech and language disorder, selective mutism and learning disorders). We included mental health conditions that are common during school age and were selected based on their relevance to the school context.

#### Severity of Co- occurring Difficulties

Severity of co-occurring difficulties was measured using the Strengths and Difficulties Questionnaire subscales conduct problems, emotional symptoms and hyperactivity- inattention of the SDQ (Goodman, 1997, 2001). The subscales peer relationship problems and prosocial behavior were not used. Participants were asked to indicate their child’s severity of co- occurring conditions on 15 SDQ- items, which were rated on a three- point Likert scale (0 = not true, 1 = somewhat true, 2 = certainly true; higher scores indicate more difficulties). The SDQ is a brief behavioral screening questionnaire with satisfactory reliability and validity of parent report (α = 0.73) (Goodman, 1997, 2001; Van Widenfelt et al., 2003). The SDQ was administered in 2017, 2018, 2020 and 2022.

#### Quality of Life

Similar to Grove et al. (2018), the Cantril Ladder (Cantril, 1965) was used to measure QoL. It was administrated from 2016 to 2022. The Cantril Ladder has good reliability and good convergent validity (Levin & Currie, 2014). Participants were asked to indicate their child’s QoL on a scale from 0 to 10, where 0 represents the worst life imaginable and 10 represents the best.

#### School Type

Participants were asked to report the current school type their child attends, with 14 educational settings provided as answer options, categorized as 1 = general education and 2 = special education school. In the Netherlands, two kinds of special education schools exist, *special education* schools (special schools as they exist in many countries), and *special primary education* schools. In the latter, the regular curriculum is taught but in a smaller class size and with extra support. These two kinds are grouped as special education schools and contrasted with general education. However, the contrast *special education* with *regular* + *special primary education* is also possible. We reported the results of those analyses in the supplemental material.

#### Autistic Traits

Autistic traits were measured by the Autism-Spectrum Quotient (AQ-short) (Hoekstra et al., 2011), administered at the time of enrollment in the NAR. Participants were invited to respond to a four-point Likert scale from 1 = totally agree to 4 = totally disagree on the 28 items that assess a fascination for numbers/patterns (5 items) and social behavioral difficulties (23 items) of their child. The social behavioral factor includes social skills (7 items), routine (4 items), switching (4 items) and imagination (8 items). Higher total scores represented higher autism severity and impaired social skills. The AQ- short has good internal consistency (Cronbach’s alpha between 0.77 and 0.86) and highly correlates with the full scale AQ (*r* between 0.93 and 0.95) (Hoekstra et al., 2011).

#### Intelligence

Participants reported their child’s most recent examined IQ test score. The seven different answer options were then recoded in seven categories: 1 = below 40, 2 = 41 to 55, 3 = 56 to 70, 4 = 71 to 85, 5 = 86 to 115, 6 = 116 to 130, 7 = above 130 and entered in the analysis as an interval variable coded from 1 to 7.

#### Age

Participants reported, prior to the current study, their child’s age in the baseline questionnaire of the NAR.

#### Gender

Participants were asked to report their child’s gender, choosing male, female or other in the baseline questionnaire of the NAR.

#### Family Socioeconomic Status (SES)

Parental educational level was used for measuring the family SES. That is because this is the largest driver of educational attainment of their children (Melby et al., 2008). The Family Profile, a 5- item questionnaire developed and used in the NAR, asking, for example, year of birth and highest educational level. Parental education level was categorized into high, middle, low (based on the highest educational level attained from either parent) based on Dutch guidelines (Centraal Bureau voor de Statistiek (CBS), 2023).

#### Year

Year was the year of the wave, 2013, 2015 to 2022.

### Procedure

Upon entering the NAR, participants received a link to fill in an online baseline questionnaire, followed by annual invitations to fill in an online questionnaire. For this study, data from the baseline questionnaire and yearly questionnaires from 2013 to 2022 were used. In 2014 there was no annual questionnaire. The baseline questionnaire covered a variety of domains, and the content of the annual questionnaires differed. Each year, participants could decide whether to participate. Only reports from children between the ages of 5 and 15, the ages at which children attend primary or secondary school, were used. The study was preregistered at the OSF/centre for open science (https://osf.io/3u9sw/).

### Statistical Analysis

This study has a longitudinal design using data collected through the NAR, including nine waves with online parental reports. Most parents (> 65%) participated in three or more waves; 15% of the parents participated only once. Co-occurring conditions were categorised into externalizing, internalizing and language- learning conditions and centred for year.

We used SPSS version 28.0 to perform hierarchical logistic analyses and stepwise multiple regression analyses. To explore the relation between the predictors and the frequency of co-occurring externalizing, internalizing and language-learning conditions a hierarchical logistic analysis with two levels was performed. For the exploration of the relation between the predictors and severity of co-occurring difficulties—emotional, behavioral and hyperactivity/inattention difficulties- as measured by the SDQ, a stepwise multiple regression analysis was performed. The same analysis was used to explore the relation between the predictors and QoL.

In the hierarchical logistic analysis analyzing the frequency and in the stepwise multiple regression analysis analyzing the severity of co-occurring conditions. We first included only school type and year as predictors in the model. In the next step, we added an interaction term between year and school type, and finally the predictors at the level of the child: autistic traits, age, gender, intelligence and family socioeconomic status. We report the results from the full model in the main text; results with fewer predictors are reported in the Supplementary Material.

In the stepwise multiple regression analysis for QoL, school type was entered first, followed by child-level co-occurring variables: externalizing, internalizing and language-learning conditions. Then we entered a two-way interaction dummy between school type and the presence of a co-occurring condition (per category). Finally, we added the predictors at the level of the child.

To ascertain that the categories of conditions did not largely overlap, we computed odds ratios for each combination and set as a criterion for keeping categories that the odds ratio was below 3. However, they were all below 1.00. Some of the other predictors in the analyses had one or more significant correlations with other predictors, but for all predictors the Variation Inflation Factor statistics were lower than the common threshold of 10 (Myers, 1990), except for the quadratic trend of year. Thus, although quadratic trend of year was pre-registered, it was not included.

For individual participants there is a perfect correlation between age and year. However, the sample was not constant over time, with participants starting at different years (waves) with different ages when they first participated. This resulted in virtually no correlation between age and year over the whole sample, see Table 2.

**Table 2:** Correlation coefficients between the independent variables

Predictors	Year	Autistic traits	Age	Gender	IQ	Family SES	Externalizing conditions	Internalizing conditions	Language- learning conditions ^c^
School type^a,c^	−.04^*^	.08^**^	−.09^**^	−.05^**^	−.43^**^	−.10^**^	.07^**^	.04^*^	.05^**^
Year		−.02	.05^**^	.06^**^	−.02	.08^**^	−.06^**^	.04	−.06^**^
Autistic traits			−.06^**^	−.08^**^	−.02	−.08^**^	.03	.11^**^	.02
Age				−.00	.04^**^	−.03	.09^**^	.08^**^	.04^*^
Gender^b, c^					−.01	.07^**^	−.04^*^	.05^**^	−.03^*^
IQ						.11^**^	.03	.02	−.06^**^
Family SES							−.08^**^	−.01	−.01
Externalizing conditions^c^								.09^**^	.13^**^
Internalizing conditions^c^									.09^**^

^*^(p < 0.05) ** (p < 0.01)

^a^Dichotomous variable with 1 for general education, 2 for special education school

^b^Dichotomous variable with 1 for male, 2 female

^c^The dichotomous variable merely indicates the existence of a correlation between the variables

There were fluctuations in the sample, with participants joining or leaving with each wave. These fluctuations in the sample result in missing values. Missing values were treated using the method Full Information Maximum Likelihood, in which missing values were not replaced or imputed. We used multiple regression models for the analyses, that way all available information is used to estimate the models.

To get an impression of how fluctuations in the sample may have impacted on our results, we also performed the analyses with a restricted sample of those participants who had participated in at least two, at least three or at least four waves. These are reported in the supplementary material.

## Results

The proportion of co-occurring conditions is presented in Table 1. Tables 3, 4, and 5 report the results of analyses examining differences in the frequency and severity of co-occurring conditions and quality of life (QoL) among autistic students across school types and years, as well as their variation over time by school type. Autistic children in special education schools were more likely to have co-occurring externalizing (B = 0.54, SE = 0.11, p < 0.001), internalizing (B = 0.53, SE = 0.21, p = 0.010), and language-learning conditions (B = 0.40, SE = 0.17, p = 0.017). For language-learning conditions this difference became more pronounced over time (*B* = 0.21, *SE* = 0.07, *p* = 0.003). The interaction term was not significant for externalizing and internalizing conditions (Table 3). Children with autism attending special education schools also exhibited more behavioral (B = 0.65, SE = 0.27, p = 0.016) and hyperactivity/inattention difficulties (B = 0.84, SE = 0.27, p = 0.002) compared to those in general education (Table 4). However, school type and all three categories of co-occurring conditions showed no overall association with QoL (Table 5). An exception was found for children with externalizing co-occurring conditions attending special education schools, who had lower QoL scores (B = −0.85, SE = 0.30, p = 0.005) than expected based on either externalizing conditions or special education school placement alone. The other interaction terms were not related to QoL (Table 5).

**Table 3:** Results of hierarchical logistic analysis exploring predictors and the frequency of co-occurring conditions

Predictors	Externalizing conditions			Internalizing conditions			Language-learning conditions		
	*B*	*SE*	Exp*(B)*	*B*	*SE*	Exp*(B)*	*B*	*SE*	Exp*(B)*
School type^a^	.54	.11	1.72^***^	.53	.21	1.70^*^	.40	.17	1.49^*^
Year	−.06	.02	.94^**^	.06	.04	1.06	−.14	.04	.87^***^
School type x Year	−.02	.04	.98	.00	.08	1.00	.21	.07	1.23^**^
Autistic traits	.01	.01	1.01	.04	.01	1.04^***^	−.01	.01	.99
Age	.10	.02	1.11^***^	.09	.04	1.09^*^	.14	.03	1.15^***^
Gender^b^	−.06	.13	.95	.35	.21	1.42	−.36	.19	.70
IQ-score	−.08	.06	.93	.13	.11	1.13	−.44	.09	.64^***^
Family SES	−.15	.09	.86	.13	.18	1.14	.14	.13	1.15
Constant	− 3.06	.73	.05^***^	− 9.31	1.38	.00^***^	− 1.79	1.04	.17
Chi^2^ (*Df* = 8)	68.37^***^			32.47^***^			82.49^***^		

^*^ (p < 0.05) **(p < 0.01) *** (p < 0.001)

^a^Dichotomous variable with 1 for general education, 2 for special education school

^b^Dichotomous variable with 1 for male, 2 female

**Table 4:** Results of stepwise multiple regression analysis exploring predictors and the severity of co-occurring difficulties

Predictors	Emotional difficulties			Behavioral difficulties			Hyperactivity/Inattention difficulties		
	Unstandardized *B*	*SE*	t-ratio	Unstandardized *B*	*SE*	t-ratio	Unstandardized *B*	*SE*	*t*-ratio
School type^a^	−.06	.32	−.20	.65^*^	.27	2.41	.84^**^	.27	3.14
Year	−.49^***^	.06	− 7.78	.46^***^	.05	8.62	−.04	.05	−.81
School type x Year	.12	.12	.95	−.25^*^	.10	− 2.42	−.20	.10	− 1.92
Autistic traits	.08^***^	.01	8.00	.01	.01	1.55	.02^*^	.01	2.35
Age	−.03	.05	−.50	−.05	.04	− 1.16	−.19^***^	.04	− 4.33
Gender^b^	1.86^***^	.26	7.21	.60^**^	.22	2.72	−.04	.22	−.18
IQ-score	.04	.13	.30	−.13	.11	− 1.18	−.10	.11	−.89
Family SES	−.04	.21	−.17	−.18	.18	− 1.02	−.59^***^	.18	− 3.36
Constant	− 3.59^*^	1.58	− 2.27	.66	1.35	.49	7.21^***^	1.22	5.92
*R*^2^	.27			.20			.11		

^*^ (p < 0.05) **(p < 0.01) *** (p < 0.001)

^a^Dichotomous variable with 1 for general education, 2 for special education school

^b^Dichotomous variable with 1 for male, 2 female

**Table 5:** Results of stepwise multiple regression analysis exploring predictors of QoL

Predictors	Unstandardized *B*	*SE*	*t*-ratio
School type^a^	−.00	.15	−.02
Externalizing conditions	.13	.15	.83
Internalizing conditions	−.32	.19	− 1.72
Language-learning conditions	.17	.16	1.05
School type x externalizing conditions	−.85^**^	.30	− 2.80
School type x internalizing conditions	−.27	.37	−.74
School type x language-learning conditions	−.61	.34	− 1.81
Year	−.01	.04	−.23
Autistic traits	−.02^***^	.01	− 3.39
Age	−.04	.03	− 1.45
Gender^b^	−.56^***^	.15	− 3.62
IQ-score	.09	.07	1.27
Family SES	−.06	.11	−.54
Constant	9.17^***^	.94	9.78
*R*^2^	.12		

^*^ (p < 0.05) **(p < 0.01) *** (p < 0.001)

^a^Dichotomous variable with 1 for general education, 2 for special education school

^b^Dichotomous variable with 1 for male, 2 female

Between 2013 and 2022, the likelihood of externalizing (B = − 0.06, SE = 0.02, p = 0.005) and language-learning conditions (B = − 0.14, SE = 0.04, p < 0.001) decreased (Table 3). Regarding severity, emotional difficulties decreased over time (B = − 0.49, SE = 0.06, p < 0.001), whereas behavioral difficulties increased (B = 0.46, SE = 0.05, p < 0.001). No significant association was found between year and hyperactivity/inattention difficulties. However, an interaction effect indicated that, over the investigated period, behavioral difficulties decreased among children in special education schools compared to those in general education (B = − 0.25, SE = 0.10, p = 0.011). This interaction was not significant for emotional or hyperactivity/inattention difficulties (Table 4). Year was not significantly associated with QoL (Table 5).

Secondly, the results of our analyses investigating the associations between the frequency and severity of co-occurring conditions, quality of life (QoL), and key characteristics of autistic youth are presented in Tables 3, 4, and 5. Older children were more likely to have externalizing (B = 0.10, SE = 0.02, p < 0.001), internalizing (B = 0.09, SE = 0.04, p = 0.028), and language-learning conditions (B = 0.14, SE = 0.03, p < 0.001). The likelihood of internalizing conditions was greater for children with higher scores on autistic traits (B = 0.04, SE = 0.01, p < 0.001). Children with lower IQ scores were more likely to have language-learning conditions (B = − 0.44, SE = 0.09, p < 0.001; Table 3).

Regarding severity, children with higher scores on autistic traits also experienced more emotional (B = 0.08, SE = 0.01, p < 0.001) and hyperactivity/inattention difficulties (B = 0.02, SE = 0.01, p = 0.019), while autistic traits were not related to behavioral difficulties. Additionally, autistic girls experienced more emotional (B = 1.86, SE = 0.26, p < 0.001) and behavioral difficulties (B = 0.60, SE = 0.22, p = 0.007) compared to boys. Gender was not associated with hyperactivity/inattention difficulties. Older children (B = − 0.19, SE = 0.04, p < 0.001) and children from higher SES families (B = − 0.59, SE = 0.18, p < 0.001) showed fewer hyperactivity/inattention difficulties compared to younger children and those from lower SES backgrounds. Age and family SES were not related to emotional or behavioral difficulties. IQ score was not related to any of the three difficulty types (Table 4).

With regard to QoL, girls (B = − 0.56, SE = 0.15, p < 0.001) and children with more autistic traits (B = − 0.02, SE = 0.01, p < 0.001) had lower QoL scores than boys or children with lower scores on autistic traits. None of the other child-level predictors were associated with QoL (Table 5).

In the additional analyses with restricted samples of participants who had participated in at least two, at least three or at least four waves, some results became nonsignificant (presumably because the smaller sample reduced our statistical power), however, the direction of almost all effects remained the same. As an example, the results of these analyses are shown in the supplemental material.

## Discussion

Our findings indicate that autistic children in special education schools, as well as older children, exhibit more co-occurring externalizing, internalizing, and language-learning conditions compared to younger children and those in general education. Across educational settings we observed a decrease in externalizing and language-learning conditions over the years, although language-learning conditions became more concentrated in special education schools. Furthermore, children with more autistic traits were more likely to have internalizing conditions, while those with lower IQ scores were more likely to have language-learning conditions. Girls and children with more autistic traits tended to exhibit more emotional difficulties. Autistic girls experienced more behavioral difficulties than boys. Additionally, older children and those from higher socioeconomic backgrounds appeared to experience fewer hyperactivity-inattention difficulties than younger children and peers from lower socioeconomic backgrounds. Hyperactivity-inattention difficulties were stable over time. Emotional difficulties decreased and behavioral difficulties increased, particularly in children enrolled in special education schools. Compared to those in general education, children in special education schools also tended to face more hyperactivity-inattention difficulties.

Neither the school type nor the year of the wave were related to QoL. Furthermore, externalizing, internalizing and language-learning conditions were all not related to QoL. However, autistic children in special education schools with externalizing co-occurring conditions reported a lower QoL than would be anticipated solely based on externalizing conditions or special education school attendance. Finally, female gender and having a higher number of autistic traits were linked to decreased QoL.

Our study aligns with earlier ones (Ledoux et al., 2020; Morningstar et al., 2017; Van den Helder et al., 2024), indicating that autistic children attending special education schools typically exhibit more co-occurring conditions and more severe behavioral and hyperactivity-inattention difficulties than their peers in general education. This may be related to the principles of inclusive education; wherein special education is only accessible for children with significant challenges. The observation of increased externalizing conditions and more severe behavioral difficulties in special education schools combined with an increase of behavioral difficulties over time, underscores the complexity of externalizing challenges in children with autism in special education schools. This complexity, in particular related to externalizing challenges, has been reported since the introduction of the inclusive policy in the Netherlands in 2014 (De Boer, 2020).

The observed increase in language-learning conditions among children enrolled in special education schools, suggests that autistic children may be referred to a special education school more often for their language learning difficulties and less because of their autism. This aligns with previous studies indicating that teachers are less positive about the inclusion of autistic children in general education with specific learning disabilities, like language-learning difficulties (De Boer et al., 2011; Krischler & Pit-ten Cate, 2020; Russell et al., 2023; Tant & Watelain, 2016).

In a poorly adapted educational environment, children with autism often display externalizing challenges, as a result of environmental distress (Green, 2019). One of the possible explanations for the observed decreases over the years in our study may be an educational environment more specifically adjusted to the educational needs arising from behavior- or hyperactivity-inattention difficulties. Our findings indicate stabilization in internalizing conditions and a decline over time in externalizing and language-learning conditions. In addition, we observed a decrease in severity of behavioral and hyperactivity-inattention difficulties among children in special education schools, aligning with the conclusion of (Simonoff et al., 2013) that special education school placement may be linked to fewer behavioral difficulties.

Autistic girls in our study experienced more behavioral difficulties than their autistic male peers. This aligns with review findings indicating that, autistic females may be presenting more externalizing challenges than autistic males (Cruz et al., 2024). Our findings may suggest that the girls in our study, compared to the boys, needed to exhibit more behavioral difficulties to become diagnosed with autism (Duvekot et al., 2017; Posserud et al., 2021).

The relatively low rates of conditions observed in our study may be attributed to the characteristics of our cohort and the specific criteria to define these conditions. The NAR includes participants from the general population, while other studies often recruit within clinics, which usually have higher rates of co-occurring conditions. Secondly, previous studies have defined co-occurring conditions based on elevated levels of symptom surveys (like the SDQ), rather than an official DSM classification (Hossain et al., 2020).

Interventions, like school placement, may have an impact QoL, either negatively or positively, based on how well the needs of an individual are met (Lord et al., 2022). In our study we did not find an association between the type of school and QoL. This may suggest that the impact of educational interventions promoting QoL depends more on the implementation of personalized adjustments, rather than the distinction between general education or special education school. Despite the importance of educational environments for optimizing autistic children’s QoL (Green, 2019), studies exploring the relationship between the learning environment and QoL are scarce (Leifler et al., 2021).

We observed that children with more autistic traits had a lower rated QoL, aligning with studies that show a negative association between autistic traits and QoL (Green et al., 2016; Oakley et al., 2021). While in previous studies (Adams et al., 2020; Green et al., 2016; Oakley et al., 2021) both autistic traits and co-occurring difficulties were negatively associated with QoL, our findings did not suggest an association between co-occurring conditions and QoL. The discrepancy might be explained by how autism-related anxiety symptoms intersect with internalizing co-occurring conditions (Adams et al., 2020).

Contrary to previous studies that did not find an association between gender and QoL in autistic children (Chiang & Wineman, 2014), our findings indicate that girls experienced lower QoL and more emotional and behavioral difficulties than boys. This difference may be explained by the social functioning domain of QoL (Menezes & Mazurek, 2021; Varni & Limbers, 2009); autistic girls tend to have more peer conflicts than autistic boys, often reflecting relational conflicts that are difficult to manage. Such conflicts may contribute to feelings of insecurity, social withdrawal and loneliness due to ending friendships (Sedgewick et al., 2019).

### Limitations

The current study was not without limitations. The sample size showed a declining trend over time limiting our ability to explore the trajectory of co- occurring conditions and difficulties in later years of our study. Additionally, sudden variations in the sample require cautious interpretation of predictor effects. The supplemental analyses focused on participants involved in two or more waves strengthened our confidence in the findings.

Furthermore, the SDQ measuring the severity of co-occurring difficulties was collected over a relatively short period of time with two interruptions. In exploring the severity trajectory of co-occurring difficulties, the impact of year could be overestimated.

In our study we used the SDQ -following Simonoff et al. (2013) and Stringer et al. (2020)—to assess the severity of co-occurring problems to examine the frequency and severity of co-occurring difficulties. We used the subscale ‘emotional problems’ for internalizing difficulties and the subscales ‘conduct problems’ and ‘hyperactivity-inattention problems’ for externalizing difficulties. However, using two SDQ subscales to assess externalizing difficulties may be suboptimal. Moreover, the SDQ lacks a subscale to assess language-learning conditions.

Our findings are based on parent ratings, which may introduce some bias particularly in subjective aspects such as QoL. Although parents can be reliable informants on their autistic children, the parental QoL scores may differ from how children will rate QoL for themselves (Stokes et al., 2017). The IQ score was reported by the parents to the best of their knowledge. While parents may strive to provide accurate information, minor discrepancies or recall errors are possible.

### Practical Considerations

The current study underscores practical considerations. Despite the shift in the western world towards inclusive education, continued investments in special education schools could contribute to the further development of these schools into centers of expertise for autistic students with co-occurring conditions. These centers of expertise could support teachers in general education with the inclusion of children with autism and co-occurring conditions. The exchange of knowledge about autism and co-occurring conditions between general and special education schools could be promoted by the inclusive policy (Roberts & Simpson, 2016; Van Kessel et al., 2021).

## Conclusion

Studies into the impact of school placement on the trajectory of co-occurring conditions and difficulties and QoL in autistic children in regular- vs- special education schools are rather limited. Our findings indicate that autistic children in general, in particular those with co-occurring conditions, are still more likely to attend special education schools. Additionally, special education schools tend to have a higher concentration of children with more severe co-occurring conditions. Attending a special education school is not necessarily associated with a lower QoL for autistic children with co-occurring conditions, suggesting that these environments may provide support that aligns with their needs.

Research may contribute to better understanding the heterogeneity of ASD and co-occurring conditions and might reveal information about the mechanisms for change in the trajectory of co-occurring conditions. More information on these mechanisms is needed to establish personalized interventions which can improve the QoL of children with autism.

## Supplementary Information

Below is the link to the electronic supplementary material.

## Data Availability

The data for this research is not available.

## Code Availability

The software code for this research is not available.
