# Supplementary material for: Co-occurring Conditions and Quality of Life in Autistic Children Attending General Education or Special Education Schools
Source: J Autism Dev Disord. 2025 May 7;56(10):3988–4000. doi: 10.1007/s10803-025-06839-3 (PMC13538233; doi:10.1007/s10803-025-06839-3)
Supplement: Supplementary file 1 [file 10803_2025_6839_MOESM1_ESM.pdf]

Co-occurring conditions and quality of life in autistic children attending general education or special education schools.

Supplemental material

The results of the full model of the hierarchical logistic regression analysis to examine the predictors and frequency of co-occurring conditions are presented in the main text. Table S1 presents the results of the analysis with school type and year as predictors. Table S2 presents the results from the analysis with school type, year and the interaction term between school type and year 2.

Table S1

Results of the Hierarchical Logistic Analysis Exploring Predictors (School Type, Year) and Frequency of Co- occurring Conditions

Predictors	Externalizing conditions			Internalizing conditions			Language-learning conditions		
	B	*SE*	Exp(*B*)	*B*	SE	Exp(*B*)	*B*	*SE*	Exp(*B*)
School type [a]	.58	.11	1.77***	.47	.19	1.59*	.45	.15	1.56**
Year	-.06	.02	.95*	.07	.04	1.07	-.11	.03	.90***
Constant	-2.12	.17	.12***	-3.56	.32	.03***	-2.94	.25	.05***
Chi² (*Df*=2)	38.78***			8.82*			22.51***		

Notes * *(p < 0.05) **(p < 0.01)*** (p < 0.001).* [a] *Dichotomous variable with 1 for general education, 2 for special education school.*

Table S2

Results of the Hierarchical Logistic Analysis Exploring Predictors (School Type, Year, Interaction Term Between School Type and Year) and Frequency of Co- occurring Conditions

Predictors	Externalizing conditions			Internalizing conditions			Language-learning conditions		
	B	*SE*	Exp(*B*)	*B*	*SE*	Exp(*B*)	*B*	*SE*	Exp(*B*)
School type [a]	.57	.11	1.78***	.46	.20	1.59*	.54	.16	1.72***
Year	-.06	.02	.95*	.07	.04	1.07	-.11	.03	.89***
School type x year	-.11	.04	.99	.01	.08	1.01	.21	.07	1.23**
Constant	-2.12	.17	.12***	-3.55	.33	.03***	-3.10	.27	.05***
Chi² (*Df*=3)	38.84			8.83**			35.59***		

Notes * *(p < 0.05) **(p < 0.01)*** (p < 0.001). [a] Dichotomous variable with 1 for general education, 2 for special education school.*

The results of the stepwise multiple regression analysis with fewer predictors examining the relation between these predictors and severity of co-occurring conditions are presented in Table S3 and S4.

Table S3

Results of the Stepwise Multiple Regression Analysis Exploring Predictors (School Type, Year) and Severity of Co- occurring Difficulties

Predictors	Emotional difficulties			Behavioral difficulties			Hyperactivity/Inattention difficulties		
	Unstandardized *B*	*SE*	*t*-ratio	Unstandardized *B*	*SE*	*t*-ratio	Unstandardized *B*	*SE*	*t*-ratio
School type [a]	.12	.24	.48	.31*	.19	1.62	.71***	.20	3.67
Year	-.49***	.07	-7.34	.50***	.05	9.62	.01	.05	.20
Constant	4.99***	.41	12.32	1.23***	.32	3.87	3.26***	.32	10.05
R^2	.10			.16			.03		

Notes * *($p < 0.05$) **($p < 0.01$)*** ($p < 0.001$).* [a] *Dichotomous variable with 1 for general education, 2 for special education school.*

Table S4

Results of the Stepwise Multiple Regression Analysis Exploring Predictors (School Type, Year, Interaction Term Between School Type and Year) and Severity of Co- occurring Difficulties

Predictors	Emotional difficulties			Behavioral difficulties			Hyperactivity/Inattention difficulties		
	Unstandardized *B*	Std error	*t*-ratio	Unstandardized *B*	Std error	*t*-ratio	Unstandardized *B*	Std error	*t*-ratio
School type [a]	-.02	.34	-.06	.77**	.26	2.94	1.14***	.27	4.24
Year	-.48***	.07	-7.03	.47***	.05	8.90	-.02	.05	-.30
School type x Year	.08	.13	.58	-.27*	.10	-2.55	-.24*	.11	-2.28
Constant	5.19***	.53	9.76	.55	.41	1.32	2.63***	.42	6.21
R^2	.10			.18			.04		

Notes * *($p < 0.05$) **($p < 0.01$)*** ($p < 0.001$). [a] Dichotomous variable with 1 for general education, 2 for special education school.*

The full results of the *stepwise multiple* regression analysis for QoL are presented in the main text. Table S5, Table S6 and Table S7 present the results of the analyses with fewer predictors.

Table S5

Results of the Stepwise Multiple Regression Analysis Exploring Predictors (School Type) of QoL

Predictors	Unstandardized *B*	*SE*	*t*-ratio
School type [a]	-.05	.14	-.40
Constant	6.58***	.23	29.05
R^2	.00		

Notes * *(p < 0.05) **(p < 0.01)*** (p < 0.001).* [a] *Dichotomous variable with 1 for general education, 2 for special education school.*

Table S6

Results of the Stepwise Multiple Regression Analysis Exploring Predictors (School Type, Externalizing Conditions, Internalizing Conditions, Language- Learning Conditions) of QoL

Predictors	Unstandardized *B*	*SE*	*t*-ratio
School type [a]	-.07	.14	-.48
Externalizing conditions	.03	.15	.20
Internalizing conditions	-.55**	.19	-2.98
Language-learning conditions	.08	.16	.51
Constant	6.64***	.25	26.44
R^2	.03		

Notes * *($p < 0.05$) **($p < 0.01$)*** ($p < 0.001$).* [a] *Dichotomous variable with 1 for general education, 2 for special education school.*

Table S7

Results of the Stepwise Multiple Analysis Exploring Predictors(School Type, Externalizing Conditions, Internalizing Conditions, Language- Learning Conditions, Interaction Terms School Type and Co- occurring Conditions) of QoL

Predictors	Unstandardized *B*	*SE*	*t*-ratio
School type [a]	.03	.14	.20
Externalizing conditions	.06	.15	.41
Internalizing conditions	-.51**	.19	-2.76
Language-learning conditions	.16	.16	.99
School type x externalizing conditions	-.83**	.31	-2.67
School type x internalizing conditions	-.25	.38	-.66
School type x language-learning conditions	-.65	.34	-1.90
Constant	6.47***	.26	25.07
R^2	.05		

Notes * *(p < 0.05) **(p < 0.01)*** (p < 0.001). [a] Dichotomous variable with 1 for general education, 2 for special education school.*

Comparison including special primary school as regular school

We also performed our analysis with special education school versus general education incl. special primary education to allow better comparison of our results with countries that do not have such thing as special primary education. These analyses are additional to the ones reported in the main text, with special education school (including special primary school) versus general education as the predictor variable.

First, we performed the hierarchical logistic regression analysis to explore the relation between our predictors and frequency of co- occurring conditions. For co-occurring externalizing and language learning conditions the results did not differ from the main analysis. Unlike the main analysis, for internalizing co- occurring conditions age was not a significant predictor. Only in the first model type of education was not related to the likelihood of co- occurring internalizing conditions, see Table S8 to S10.

Table S8

Results of the Hierarchical Logistic Analysis Exploring Predictors (School Type, Year) and Frequency of Co- occurring Conditions, with Special Education School versus General Education incl. Special Primary School as Predictor Variable

Predictors	Externalizing conditions			Internalizing conditions			Language-learning conditions		
	B	*SE*	Exp(*B*)	*B*	*SE*	Exp(*B*)	*B*	*SE*	Exp(*B*)
School type [a]	.52	.11	1.68***	.37	.19	1.44	.60	.15	1.82***
Year	-.05	.02	.95*	.07	.04	1.07	-.10	.03	.90**
Constant	-.197	.16	.14***	-3.36	.29	.04***	-3.12	.24	.04***
Chi² (*Df*=2)	32.85***			6.47*			29.69***		

Notes * *(p < 0.05) **(p < 0.01)*** (p < 0.001).* [a] *Dichotomous variable with 1 for general education, 2 for special education school.*

Table S9

Results of the Hierarchical Logistic Analysis Exploring Predictors (School Type, Year, Interaction Term Between School Type and Year) and Frequency of Co- occurring Conditions, with Special Education School versus General Education incl. Special Primary School as Predictor Variable

Predictors	Externalizing conditions			Internalizing conditions			Language-learning conditions		
	B	*SE*	Exp(*B*)	*B*	*SE*	Exp(*B*)	*B*	*SE*	Exp(*B*)
School type [a]	.52	.11	1.68***	.39	.19	1.48*	.69	.15	2.00***
Year	-.05	.02	.95*	.07	.04	1.07	-.12	.03	.89***
School type x year	.01	.04	1.01	-.06	.08	.94	.22	.07	1.24***
Chi² (*Df*=3)	32.92***			7.20			40.89***		

Notes * *($p < 0.05$) **($p < 0.01$)*** ($p < 0.001$). [a] Dichotomous variable with 1 for general education, 2 for special education school.*

Table S10

Results of the Hierarchical Logistic Analysis Exploring Predictors and Frequency of Co- occurring Conditions, with Special Education School versus General Education incl. Special Primary School as Predictor Variable

Predictors	Externalizing conditions			Internalizing conditions			Language-learning conditions		
	B	*SE*	Exp(*B*)	*B*	*SE*	Exp(*B*)	*B*	*SE*	Exp(*B*)
School type [a]	.42	.11	1.53***	.41	.20	1.50*	.51	.16	1.66**
Year	-.06	.02	.94**	.06	.04	1.06	-.14	.04	.87***
School type x Year	-.00	.04	1.00	-.07	.08	.93	.20	.07	1.22**
Autistic traits	.01	.01	1.01	.04	.01	1.04***	-.01	.01	.99

Notes * *($p < 0.05$) **($p < 0.01$)*** ($p < 0.001$). [a] Dichotomous variable with 1 for general education, 2 for special education school.*

Table S10 continued

Predictors	Externalizing conditions			Internalizing conditions			Language-learning conditions		
	B	*SE*	Exp(*B*)	*B*	*SE*	Exp(*B*)	*B*	*SE*	Exp(*B*)
Age	.09	.02	1.09***	.08	.04	1.08	.13	.03	1.14***
Gender [b]	-.06	.13	.95	.36	.21	1.43	-.35	.19	.71
IQ-score	-.011	.06	.89	.08	.11	1.08	-.44	.09	.64***
Family SES	-.16	.09	.85	.13	.18	1.14	.14	.13	1.15
Constant	-2.51	.70	.08***	-8.79	1.34	.00***	-1.76	1.01	.17
Chi² (*Df*=8)	59.39***			29.96***			86.08***		

Notes * *($p < 0.05$) **($p < 0.01$)*** ($p < 0.001$).* [b] *Dichotomous variable with 1 for male, 2 female.*

Then we again performed a stepwise multiple regression analysis to explore the relation between the predictors and severity of co- occurring difficulties - emotional, behavioral and hyperactivity/ inattention difficulties. The results for emotional and hyperactivity/ inattention were similar to those of the main analysis. Unlike the main analysis, school type and the interaction between school type and year were both not related to severity of behavioral problems, see Table S11 to S13.

Table S11

Results of the Stepwise Multiple Regression Analysis Exploring Predictors (School Type, Year) and Severity of Co- occurring Difficulties, with Special Education School versus General Education incl. Special Primary School as Predictor Variable

Predictors	Emotional difficulties			Behavioral difficulties			Hyperactivity/Inattention difficulties		
	Unstandardized *B*	*SE*	*t*-ratio	Unstandardized *B*	*SE*	*t*-ratio	Unstandardized *B*	*SE*	*t*-ratio
School type [a]	-.33	.25	-1.31	.08	.20	.40	.45*	.20	2.23
Year	-.50***	.07	-7.48	.50***	.05	9.50	.01	.05	.17
Constant	5.64***	.39	14.31	1.59***	.31	5.12	3.70***	.332	11.61
R^2	.11			.16			.01		

Notes * *($p < 0.05$) **($p < 0.01$)*** ($p < 0.001$).* [a] *Dichotomous variable with 1 for general education, 2 for special education school .*

Table S12

Results of the Stepwise Multiple Regression Analysis Exploring Predictors (School Type, Year, Interaction Term Between School Type and Year) and Severity of Co- occurring Difficulties, with Special Education School versus General Education incl. Special Primary School as Predictor Variable

Predictors	Emotional difficulties			Behavioral difficulties			Hyperactivity/Inattention difficulties		
	Unstandardized *B*	*SE*	*t*-ratio	Unstandardized *B*	*SE*	*t*-ratio	Unstandardized *B*	*SE*	*t*-ratio
School type [a]	-.48	.34	-1.39	.38	.27	1.39	.84**	.28	3.04
Year	-.49***	.07	-7.04	.47***	.05	8.75	-.02	.06	-.38
School type x Year	.09	.14	.63	-.18	.11	-1.62	-.24*	.11	-2.06
Constant	5.84***	.51	11.49	1.18**	.40	2.95	3.17***	.41	7.74
R^2	.11			.16			.02		

Notes * *($p < 0.05$) **($p < 0.01$)*** ($p < 0.001$).* [a] *Dichotomous variable with 1 for general education, 2 for special education school .*

Table S13

Results of the Stepwise Multiple Regression Analysis Exploring Predictors and Severity of Co- occurring Difficulties, with Special Education School versus General Education incl. Special Primary School as Predictor Variable

Predictors	Emotional difficulties			Behavioral difficulties			Hyperactivity/Inattention difficulties		
	Unstandardized *B*	*SE*	*t*-ratio	Unstandardized *B*	*SE*	*t*-ratio	Unstandardized *B*	*SE*	*t*-ratio
School type [a]	-.28	.32	-.87	.36	.27	1.33	.75**	.27	2.78
Year	-.50***	.06	-7.89	.46***	.05	8.49	-.05	.05	-.90
School type x Year	.04	.13	.30	-.19	.11	-1.73	-.21	.11	-1.87
Autistic traits	.08***	.01	8.02	.02	.01	1.71	.02*	.01	2.55
Age	-.02	.05	-.39	-.05	.04	-1.24	-.20***	.04	-4.62
Gender [b]	1.82***	.26	7.02	.60**	.22	2.70	-.02	.22	-.10
IQ-score	-.01	.13	-.09	-.16	.11	-1.53	-.13	.11	-1.23
Family SES	-.05	.21	-.24	-.20	.18	-1.13	-.61***	.18	-3.48
Constant	-3.02*	1.53	-1.97	1.30	1.31	.99	6.37***	1.30	4.89
R^2	.27			.19			.11		

Notes * *($p < 0.05$) **($p < 0.01$)*** ($p < 0.001$).* [a] *Dichotomous variable with 1 for general education, 2 for special education school .*[b] *Dichotomous variable with 1 for male, 2 female.*

Finally, we also repeated the stepwise multiple regression analysis to assess the predictors for QoL of children with autism. Unlike the main analysis, the interaction between school type and externalizing co- occurring conditions was not related to QoL. The results for the other predictors did not differ from the main analysis, see Table S14 to S17.

Table S14

Results of the Stepwise Multiple Regression Analysis Exploring Predictors (School Type) of QoL, with Special Education School versus General Education incl. Special Primary School as Predictor Variable

Predictors	Unstandardized *B*	*SE*	*t*-ratio
School type [a]	-.09	.14	-.66
Constant	6.60***	.21	31.88
R^2	.00		

Notes * *($p < 0.05$) **($p < 0.01$)*** ($p < 0.001$).* [a] *Dichotomous variable with 1 for general education, 2 for special education school.*

Table S15

Results of the Stepwise Multiple Regression Analysis Exploring Predictors (School Type, Externalizing Conditions, Internalizing Conditions, Language- Learning Conditions) of QoL, with Special Education School versus General Education incl. Special Primary School as Predictor Variable

Predictors	Unstandardized *B*	*SE*	*t*-ratio
School type [a]	-.13	.14	-.95
Externalizing conditions	.04	.15	.26
Internalizing conditions	-.55**	.19	-2.99

Language-learning conditions	.10	.16	.63
Constant	6.61***		
R^2	.03		

Notes * *($p < 0.05$) **($p < 0.01$)*** ($p < 0.001$).* [a] *Dichotomous variable with 1 for general education, 2 for special education school.*

Table S16

Results of the Stepwise Multiple Regression Analysis Exploring Predictors(School Type, Externalizing Conditions, Internalizing Conditions, Language- Learning Conditions, Interaction Terms School Type and Co- occurring Conditions) of QoL, with Special Education School versus General Education incl. Special Primary School as Predictor Variable

Predictors	Unstandardized *B*	*SE*	*t*-ratio
School type [a]	-.05	.14	-.35
Externalizing conditions	.03	.15	.21
Internalizing conditions	-.54**	.19	-2.89
Language-learning conditions	.14	.17	.82
School type x externalizing conditions	-.53	.31	-1.70
School type x internalizing conditions	-.13	.37	-.35
School type x language-learning conditions	-40	.33	-1.23
Constant	6.61***	.24	27.35
R^2	.04		

Notes * *($p < 0.05$) **($p < 0.01$)*** ($p < 0.001$). [a] Dichotomous variable with 1 for general education, 2 for special education school.*

Table S17

Results of the Stepwise Multiple Regression Analysis Exploring Predictors of QoL, with Special Education School versus General Education incl. Special Primary School as Predictor Variable

Predictors	Unstandardized *B*	*SE*	*t*-ratio
School type [a]	-.09	.15	-.61
Externalizing conditions	.10	.15	.65
Internalizing conditions	-.34	.19	-1.84
Language-learning conditions	.15	.16	.93
School type x externalizing conditions	-.58	.30	-1.92
School type x internalizing conditions	-.19	.37	-.52
School type x language-learning conditions	-.40	.32	-1.24
Year	-.01	.04	-.32
Autistic traits	-.02***	.01	-3.42
Age	-.04	.03	-1.45
Gender [b]	-.57***	.16	-3.70
IQ-score	.10	.07	1.30
Family SES	-.06	.12	-.53
Constant	9.36***	.90	10.46
R^2	.11		

Notes * *($p < 0.05$) **($p < 0.01$)*** ($p < 0.001$).* [a] *Dichotomous variable with 1 for general education, 2 for special education school .*[b] *Dichotomous variable with 1 for male, 2 female.*

Comparison participants with more than one wave of participation

The data stems from a panel that changed over time – some respondents stopped participating while others joined at a later moment. This introduces a risk that trends over time are actually due to changes in the composition of the panel. One way to mitigate this risk, though not eliminate it, is to restrict the analyses to those respondents who participated in multiple waves, ascertaining that from one wave to the next a majority of participants remained in the sample. We therefore repeat our analyses with only those respondents who participated in at least two waves.

Frequency of co- occurring conditions

First, we again performed hierarchical logistic analyses in which we predicted the frequency of co- occurring conditions for respondents who participated more than 1 wave, more than 2 waves and finally for those participating more than 3 waves. The results of the full models are presented below and shown in Table S18, S19 and S20.

School type

Similar to the main analysis, autistic children attending special education schools were more likely to have co- occurring externalizing (*B*=0.51, *SE*=0.11, *p*<0.001/ *B*=0.76, *SE*=0.13, *p*<0.001/ *B*=0.58, *SE*=0.15, *p*<0.001)[1], and language learning conditions (*B*=0.37, *SE*=0.17, *p*=0.027/ *B*=0.58, *SE*=0.19, *p*=0.002/ *B*=0.82, *SE*=0.23, *p*<0.001) than their peers in general education. Contrary to main – and 2 to 9 waves- analysis, internalizing conditions did not reach significance in the analysis with more than 3 or 4 waves involvement (*B*=0.51, *SE*=0.21, *p*=0.015/ *B*=0.45, *SE*=0.25, *p*=0.069/ *B*=0.38, *SE*=0.28, *p*=0.170).

Year

[1] Here, and for the other effects, the first set of statistical outcomes refers to the analysis with only those who participated in more than 1 wave, the second those who participated in more than 2 waves, and the last those who participated in more than 3 waves.

Just like in the main analyses, the likelihood for externalizing (*B*=-0.47, *SE*=0.02, *p*=0.041) conditions (*B*=-0.13, *SE*=0.04, *p*<0.001) decreased between 2013 and 2022 for the participants with more than 1 wave involvement, however not for those with more than 2 (*B*=-0.25, *SE*=0.03, *p*=0.378) or 3 waves of involvement (*B*=-0.01, *SE*=0.04, *p*=0.859). Just like the main analysis, the internalizing conditions were not significant associated with year when participants participated more than 1 wave (*B*=0.08, *SE*=0.04, *p*=0.052), however, the likelihood increased between 2013 and 2022 for those with more than 2 waves (*B*=0.12, *SE*=0.05, *p*=0.010) or more than 3 waves involvement (*B*=0.16, *SE*=0.06, *p*=0.012). Alike the main analysis, the likelihood for language- learning conditions was decreased in the investigated period for participants with more than 1 wave (*B*=-0.13, *SE*=0.04, *p*<0.001) or more than 2 waves involvement (*B*=-0.13, *SE*=0.04, *p*=0.005), however not for participants involved in more than 3 waves (*B*=-0.17, *SE*=0.06, *p*=0.779).

Interaction between school type and year

Alike the main analysis, for externalizing conditions, the interaction term between school type and year did not reach significance in the analysis for the involvement in more than 1 wave (*B*=-0.03, *SE*=0.05, *p*=0.534), however it did in the analysis for more than 2 waves (*B*=-0.14, *SE*=0.06, *p*=0.010) or more than 3 waves involvement (*B*=-0.14, *SE*=0.07, *p*=0.039). Similar to the main analysis, for internalizing conditions the interaction term did not reach significance in the additional analyses. The analyses showed the mirror image occurred for language- learning conditions (*B*=0.20, *SE*=0.07, *p*=0.005/ *B*=0.16, *SE*=0.08, *p*=0.065/ *B*=0.17, *SE*=0.11, *p*=0.117).

Predictors at child's level

Compared to the main analysis, the autistic traits' contribution to the likelihood for externalizing, internalizing and language- learning conditions did not change in these additional analyses.

Alike the main analysis, older autistic children were more likely to have externalizing conditions in these additional analyses (*B*=0.10, *SE*=0.02, $p<0.001$/ *B*=0.12, *SE*=0.03, $p<0.001$/ *B*=0.11, *SE*=0.03, *p*=0.002). For internalizing conditions age was a significant predictor in the analysis with more than 1 waves involvement (*B*=0.08, *SE*=0.04, *p*=0.043), however it was not in the analyses with more than 2 waves (*B*=0.05, *SE*=0.05, *p*=0.322) or more than 3 waves involvement (*B*=-0.03, *SE*=0.06, *p*=0.567). In the analysis with more than 1 wave (*B*=0.14, *SE*=0.03, $p<0.001$) or more than 2 waves involvement (*B*=0.16, *SE*=0.04, $p<0.001$), older children were language- learning conditions to have language- learning conditions, alike the main analysis. However, age did not significantly contribute to the likelihood for language-learning conditions in the analysis with more than 3 waves involvement.

In these additional analyses and similar to the main analysis, gender nor socioeconomic status were significantly associated with the presence of internalizing or language- learning conditions. Contrary to the main analysis, in the analysis with more than 3 waves involvement, younger autistic children were more likely to have externalizing conditions (*B*=-0.37, *SE*=0.17, *p*=0.036). Alike the main analysis, socioeconomic status did not significantly contribute the likelihood of externalizing conditions in these additional analyses.

For the likelihood of externalizing or internalizing conditions, and similar to the main analysis, intelligence score was no significant predictor in these additional analyses. Alike the main analysis, autistic children with lower IQ- scores were more likely to have language-learning conditions (*B*=-0.45, *SE*=0.09, $p<0.001$/ *B*=-0.48, *SE*=0.10, $p<0.001$/ *B*=-0.48, *SE*=0.11, $p<0.001$).

Severity of co- occurring difficulties

We again performed stepwise multiple regression analyses in which we predicted the severity of co- occurring difficulties for respondents who participated more than 1 wave, more than 2 waves and finally for those participating more than 3 waves. Nothing changed in the regression analysis for participants who participated in more than 1 wave. They were not included in the analysis due to missings in the combination of variables in the analysis. Therefore the results of the analysis with more than 2 waves, and more than 3 waves involvement (full models) are presented below and shown in Table S21 and S22.

School type

Similar to the main analysis, school type only predicted severity of behavioral (B=0.70, SE=0.28, p=0.013/ B=0.86, SE=0.30, p=0.004)[2] and hyperactivity- inattention difficulties (B=0.94, SE=0.28, p<0.001/ B=1.00, SE=0.31, p<0.001). For severity of emotional difficulties, contrary to the main analysis, the direction of the parameter estimate changed from positive to negative.

Year

The results of the additional analyses did not differ from the main analysis for all the three types of difficulties.

Interaction term between school type and year

Alike the main analysis, the interaction term between school type did not predict severity of emotional difficulties, however the direction of the parameter estimate changed in the analysis with more than 3 waves involvement (B=-0.06, SE=0.18, p=0.748). Similar to the main analysis, autistic children attending special education schools exhibited a decrease in behavioral difficulties (B=-0.33, SE=0.12, p=0.007/(B=-0.50, SE=0.15, p<0.001) between 2013 and 2022, compared to their autistic peers attending general education. Contrary to the

[2] Here, and for the other effects, the first set of statistical outcomes refers to the analysis with only those who participated in more than 2 waves, the second those who participated in more than 3 waves.

main analysis, autistic children in special education schools exhibited a decrease in hyperactivity- inattention difficulties (*B*=-0.29, *SE*=0.12, *p*=0.016/(*B*=-0.44, *SE*=0.16, *p*=0.005).

Predictors at child's level

Children with higher scores on autistic traits exhibited more severe emotional (*B*=0.09, *SE*=0.01, *p*<0.001/ *B*=0.09, *SE*=0.01, *p*<0.001) and hyperactivity- inattention difficulties (*B*=0.03, *SE*=0.01, *p*=0.007/ *B*=0.02, *SE*=0.01, *p*=0.020). Autistic traits remained unrelated to behavioral difficulties.

Similar to the main analysis, older autistic children (*B*=-0.15, *SE*=0.05, *p*<0.001/ *B*=-0.14, *SE*=0.05, *p*=0.009) and those from higher SES families (*B*=-0.57, *SE*=0.19, *p*=0.003/ *B*=-0.73, *SE*=0.21, *p*<0.001) experienced fewer hyperactivity- inattention difficulties than younger and lower SES autistic children. Age and family SES remained unrelated to severity of emotional and behavioral difficulties.

Just like in the main analysis, autistic girls experienced more emotional difficulties (*B*=2.02, *SE*=0.27, *p*<0.001/ *B*=2.03, *SE*=0.31, *p*<0.001) compared to autistic boys. Similar to the main analysis gender was a predictor for severity of behavioral difficulties (*B*=0.57, *SE*=0.24, *p*=0.016) in the analysis with more than more than 2 waves involvement , however it was not in the analysis with more than 3 waves involvement (*B*=0.43, *SE*=0.25, *p*=0.086). Compared to the main analysis, the results for gender did not change.

In comparison with the main analysis, intelligence score remained unrelated to severity of all the three types of co- occurring difficulties, however for emotional difficulties the direction of the parameter estimate changed from positive to negative.

QoL

Finally, we again performed stepwise multiple regression analyses in which we predicted the QoL for respondents who participated more than 1 wave, more than 2 waves and

finally for those participating more than 3 waves. Similar to the previous analysis, nothing changed in the regression analysis for participants who participated in more than 1 wave. They were not included in the analysis due to missings in the combination of variables in the analysis. Therefore the results of the analysis with more than 2 waves, and more than 3 waves involvement (full models) are presented below and shown in Table S23 and S24.

School type

In comparison to the main analysis, type of education remained unrelated to QoL, however the direction of the parameter estimate changed from negative to positive.

Co- occurring conditions

The presence of externalizing, internalizing or language- learning conditions was not significantly associated with QoL, alike the main analysis.

Interaction term between type of education and co- occurring conditions

Similar to the main analysis, autistic children with externalizing conditions in special education schools had lower QoL- scores (B=-0.81, SE=0.32, p=0.012/ B=-1.09, SE=0.37, p=0.003) than would have been expected from special education school attendance or the presence of externalizing conditions alone. Alike the main analysis, the interaction terms between school type and internalizing and language- learning conditions were not associated with QoL.

Year

Year remained unrelated to QoL, however compared to the main analysis, the direction of the parameter estimate changed from negative to positive.

Predictors at child's level

Compared to the main analysis, the predictors at the level of the child did not change in the additional analysis. Still, only autistic traits (B=-0.03, SE=0.01, p<0.001/ B=-0.02,

SE=0.01, *p*=0.026) and gender (*B*=-0.51, *SE*=0.17, *p*=0.002/ *B*=-0.56, *SE*=0.20, *p*=0.005) were related to QoL.

Concluding, results for this subsample were much the same as those in the main analysis, with some effects switching from significant to not significant but with almost all the parameter estimate in the same direction. The latter can naturally be explained as a consequence of reduced power through the smaller sample.

Table S18

Results of the Hierarchical Logistic Analysis Exploring Predictors and Frequency of Co- occurring Conditions for Participants with more than 1 Wave Involvement

Predictors	Externalizing conditions			Internalizing conditions			Language-learning conditions		
	B	*SE*	Exp(*B*)	*B*	*SE*	Exp(*B*)	*B*	*SE*	Exp(*B*)
School type [a]	.51	.11	1.67***	.51	.21	1.66*	.37	.17	1.46
Year	-.05	.02	.95*	.08	.04	1.08	-.13	.04	.88***
School type x Year	-.03	.05	.98	-.00	.08	1.00	.20	.07	1.22*
Autistic traits	.01	.01	1.01	.04	.01	1.04***	-.01	.01	.99
Age	.10	.02	1.11***	.08	.04	1.09*	.14	.03	1.15***
Gender [b]	-.04	.13	.96	.37	.21	1.45	-.33	.19	.72
IQ-score	-.08	.06	.93	.13	.11	1.14	-.45	.09	.64***
Family SES	-.15	.09	.86	.13	.18	1.14	.13	.13	1.14
Constant	-2.98	.73	.05***	-9.24	1.38	.00***	-1.64	1.05	.20
*Chi*² (*Df*=8)	58.85***			33.45***			75.95***		

Notes * *($p < 0.05$) **($p < 0.01$)*** ($p < 0.001$).* [a] *Dichotomous variable with 1 for general education, 2 for special education school .*[b] *Dichotomous variable with 1 for male, 2 female.*

Table S19

Results of the Hierarchical Logistic Analysis Exploring Predictors and Frequency of Co- occurring Conditions for Participants with more than 2 Waves Involvement

Predictors	Externalizing conditions			Internalizing conditions			Language-learning conditions		
	B	*SE*	Exp(*B*)	*B*	*SE*	Exp(*B*)	*B*	*SE*	Exp(*B*)
School type [a]	.76	.13	2.14***	.45	.25	1.56	.58	.19	1.78**
Year	-.03	.03	.98	.12	.05	1.31*	-.13	.04	.88**
School type x Year	-.14	.06	.87*	-.00	.09	1.00	.15	.08	1.17
Autistic traits	.01	.01	1.01	.04	.01	1.04***	-.01	.01	.99
Age	.12	.03	1.13***	.05	.05	1.05*	.16	.04	1.17***
Gender [b]	-.07	.15	.93	.29	.25	1.33	-.39	.22	.67
IQ-score	-.09	.07	.91	.14	.13	1.15	-.48	.10	.62***
Family SES	-.18	.11	.84	.29	.22	1.33	.04	.15	1.04
Constant	-3.42	.85	.03***	-9.57	1.63	.00***	-1.65	1.19	.19
*Chi*² (*Df*=8)	72.82***			31.45***			68.09***		

Notes * *(p < 0.05) **(p < 0.01)*** (p < 0.001).* [a] *Dichotomous variable with 1 for general education, 2 for special education school .*[b] *Dichotomous variable with 1 for male, 2 female.*

Table S20

Results of the Hierarchical Logistic Analysis Exploring Predictors and Frequency of Co- occurring Conditions for Participants with more than 3 Waves Involvement

Predictors	Externalizing conditions			Internalizing conditions			Language-learning conditions		
	B	*SE*	Exp(*B*)	*B*	*SE*	Exp(*B*)	*B*	*SE*	Exp(*B*)
School type [a]	.58	.15	1.79***	.38	.28	1.47	.82	.23	2.28***
Year	-.01	.04	.99	.16	.06	1.17*	-.02	.06	.98
School type x Year	-.14	.07	.87*	.09	.11	1.09	.17	1.07	1.18
Autistic traits	.01	.01	1.01	.04	.01	1.04**	-.01	.01	1.00
Age	.11	.03	1.11**	-.03	.06	.97	.09	.05	1.09
Gender [b]	-.37	.17	.69*	.37	.29	1.44	-.35	.26	.71
IQ-score	-.07	.08	.93	.16	.14	1.18	-.48	.11	.62***
Family SES	-.17	.13	.84	.44	.27	1.55	.35	.20	1.42
Constant	-2.57	1.00	.08*	-8.64	1.94	.00***	-2.33	1.52	.10
*Chi*² (*Df*=8)	43.56***			22.53**			51.44***		

Notes * *(p < 0.05) **(p < 0.01)*** (p < 0.001).* [a] *Dichotomous variable with 1 for general education, 2 for special education school .*[b] *Dichotomous variable with 1 for male, 2 female.*

Table S21

Results of the Stepwise Multiple Regression Analysis Exploring Predictors and Severity of Co- occurring Difficulties, for Participants with more than 2 Waves Involvement

Predictors	Emotional difficulties			Behavioral difficulties			Hyperactivity/Inattention difficulties		
	Unstandardized *B*	Std error	*t*-ratio	Unstandardized *B*	Std error	*t*-ratio	Unstandardized *B*	Std error	*t*-ratio
School type [a]	.06	.33	.19	.70*	.20	2.48	.94***	.28	3.33
Year	-.44***	.07	-6.22	.42***	.06	6.98	-.04	.06	-.73
School type x Year	.01	.14	.07	-.33**	.12	-2.72	-.29*	.12	-2.43
Autistic traits	.09***	.01	8.00	.01	.01	1.41	.03**	.01	2.70
Age	-.04	.05	-.72	-.04	.05	-.86	-.15***	.05	-3.27
Gender [b]	2.02***	.27	7.42	.57*	.24	2.42	-.07	.24	-.31
IQ-score	.10	.13	.71	-.19	.12	-1.63	-.09	.12	-.74
Family SES	-.07	.22	-.24	-.19	.19	-1.00	-.57***	.19	-3.48
Constant	-4.37**	1.67	-2.67	.90	1.44	.62	4.90**	1.44	3.04
R^2	.27			.16			.11		

Notes * *($p < 0.05$) **($p < 0.01$)*** ($p < 0.001$).* [a] *Dichotomous variable with 1 for general education, 2 for special education school .*[b] *Dichotomous variable with 1 for male, 2 female.*

Table S22

Results of the Stepwise Multiple Regression Analysis Exploring Predictors and Severity of Co- occurring Difficulties, for Participants with more than 3 Waves Involvement

Predictors	Emotional difficulties			Behavioral difficulties			Hyperactivity/Inattention difficulties		
	Unstandardized *B*	Std error	*t*-ratio	Unstandardized *B*	Std error	*t*-ratio	Unstandardized *B*	Std error	*t*-ratio
School type [a]	.08	.36	.22	.86**	.30	2.91	1.00**	.31	3.25
Year	-.40***	.09	-4.37	.39***	.08	5.20	-.09	.08	-1.11
School type x Year	-.06	.18	-.32	-.50***	.15	-3.39	-.44**	.16	-2.82
Autistic traits	.09***	.01	7.73	.01	.01	.62	.02*	.01	2.34
Age	-.02	.06	-.31	-.09	.05	-1.75	-.14**	.05	-263
Gender [b]	2.03***	.31	6.54	.43*	.25	1.72	-.28	.26	-1.04
IQ-score	.05	.15	.30	-.22	.12	-1.85	-.15	.13	-1.19
Family SES	-.13	.25	-.53	-.28	.20	-1.42	-.73***	.21	-3.50
Constant	-4.69*	1.92	-2.44	2.43	1.56	1.56	5.84***	1.63	3.58
R^2	.26			.16			.14		

Notes * *($p < 0.05$) **($p < 0.01$)*** ($p < 0.001$).* [a] *Dichotomous variable with 1 for general education, 2 for special education school .*[b] *Dichotomous variable with 1 for male, 2 female.*

Table S23

Results of the Stepwise Multiple Regression Analysis Exploring Predictors of QoL, for Participants with more than 2 Waves Involvement

Predictors	Unstandardized *B*	*SE*	*t*-ratio
School type [a]	.03	.16	.18
Externalizing conditions	.12	.16	.76
Internalizing conditions	-.22	.20	-1.11
Language-learning conditions	.18	.17	1.10
School type x externalizing conditions	-.81*	.32	-2.54
School type x internalizing conditions	.00	.39	.00
School type x language-learning conditions	-.49	.35	-1.41
Year	.01	.04	.19
Autistic traits	-.03***	.01	-3.91
Age	-.06	.03	-1.77
Gender [b]	-.51**	.17	-3.08
IQ-score	.10	.08	1.30
Family SES	-.08	.12	-.65
Constant	9.63***	1.00	9.63
R^2	.13		

Notes * *($p < 0.05$) **($p < 0.01$)*** ($p < 0.001$). [a] Dichotomous variable with 1 for general education, 2 for special education school .[b] Dichotomous variable with 1 for male, 2 female.*

Table S24

Results of the Stepwise Multiple Regression Analysis Exploring Predictors of QoL, for Participants with more than 3 Waves Involvement

Predictors	Unstandardized *B*	*SE*	*t*-ratio
School type [a]	.12	.19	.63
Externalizing conditions	.08	.18	.47
Internalizing conditions	-.15	.22	-.69
Language-learning conditions	.30	.20	1.52
School type x externalizing conditions	-1.09**	.37	-2.97
School type x internalizing conditions	-.45	.43	-1.05
School type x language-learning conditions	-.71	.41	-1.75
Year	.02	.05	.49
Autistic traits	-.02*	.01	-2.25
Age	-.04	.04	-1.10
Gender [b]	-.56**	.20	-2.81
IQ-score	.12	.08	1.43
Family SES	-.04	.14	-.32
Constant	8.32***	1.13	7.34
R^2	.12		

Notes * *($p < 0.05$) **($p < 0.01$)*** ($p < 0.001$).* [a] *Dichotomous variable with 1 for general education, 2 for special education school .*[b] *Dichotomous variable with 1 for male, 2 female*
